# Non-invasive quantification of corneal vascularization using anterior segment optical coherence tomography angiography

**DOI:** 10.1038/s41598-024-52598-z

**Published:** 2024-01-24

**Authors:** Julia Aschauer, Michal Klimek, Ruth Donner, Jan Lammer, Philipp Roberts, Markus Schranz, Gerald Schmidinger

**Affiliations:** Department of Ophthalmology and Optometry, Medical University of Vienna, Spitalgasse 23, 1090 Vienna, Austria

**Keywords:** Eye diseases, Corneal diseases

## Abstract

The presence of corneal vascularization (CV) interferes with the angiogenic and immune privilege of the cornea, risking rejection in eyes following keratoplasty. Pre-operative (lymph)-angioregression is a promising therapeutic approach, but objective monitoring by non-invasive CV imaging is needed. The purpose of this study was to investigate anterior-segment optical coherence tomography angiography (AS-OCTA) for CV visualization and quantification, and to show its superiority over slit-lamp photography in high-risk eyes scheduled for keratoplasty. This institutional pilot study included 29 eyes of 26 patients (51 ± 16 years, 8 female) with significant CV scheduled for keratoplasty that were imaged by slit-lamp photography (Zeiss SL 800) and AS-OCTA (Zeiss Plex Elite 9000). After manual corneal layer segmentation correction, CV maximum/relative depth was measured with the inbuilt software. Slit-lamp photographs and AS-OCTA images were compared for visualization of vascular details. Angiotool software allowed a semi-automated determination of CV-related parameters in the vascular complex of AS-OCTA images. The predominant causes of CV were the herpes simplex virus keratitis (n = 7) and chemical burn (n = 4). Visualization of vascular morphology in AS-OCTA was superior to slit-lamp photography in all except one eye. Vascular metrics including total vessel length, number of junctions/endpoints, junction density, lacunarity, and vessel area/density were defined using Angiotool, with CV depth localization despite scarring and opacification. AS-OCTA proved effective for angioregressive treatment monitoring. AS-OCTA enables non-invasive and objective three-dimensional visualization of corneal vascularization superior to slit-lamp photography, and could be a precious tool for monitoring angioregressive preconditioning prior to keratoplasty.

## Introduction

The structure of the healthy human cornea is unique as it is completely avascular and alymphatic, which enables ideal light transmission and an optical pathway through total transparency.

This “(lymph-)angiogenic privilege” derives from a delicate dynamic balance between pro- and anti-angiogenic, as well as immune-modulatory, factors in the cornea and the adjacent aqueous humor (e.g., vascular endothelial growth factor (VEGF))^1,2^, and is the foundation of the cornea’s immune privilege.

Inflammation, trauma, chemical injury, or infection may initiate an inflammatory response which can lead to concurrent sprouting of blood and lymphatic vessels from pre-existing limbal arcuate structures^3^. Corneal vascularization (CV) is usually accompanied by significant scarring and opacification, where corneal allograft transplantation is an important sight-saving surgical therapy. However, in the presence of CV, these eyes are exposed to a significantly higher risk of allograft failure and rejection^4,5^, which correlates with the depth and extent of corneal vessels^6^.

Therefore, a constant endeavor and as yet unmet clinical need when planning penetrating keratoplasty (PK) is to provide the optimum environment at the recipient site of hosts at high risk due to stromal CV. A variety of strategies have been proposed for pre-operative “(lymph-)angioregression”, including the application of topical, stromal or subconjunctival anti-VEGF agents, with or without additional mechanical occlusive procedures such as fine-needle diathermy^7–9^, argon laser- or cryo-coagulation, or the recently introduced peripheral corneal crosslinking technique, which might show additional regressive effects on lymphatic vessels^10,11^.

However, a key requirement for estimating stratification and the risk to the host, as well as for a comprehensible monitoring of (lymph-)angioregressive treatments prior to PK, is the ability to morphologically and quantitatively assess CV with objective and reproducible imaging techniques.

Today, analyzing photographic images of the cornea acquired with slit-lamp photography is the most commonly used CV imaging method. It serves as a rapid, straightforward documentation method, but fine capillary details may not be detectable, especially in the presence of scarring. Anterior segment angiography techniques, including fluorescein angiography (FA) and indocyanine green angiography (ICGA), provide a much more accurate delineation of the vascular network of the cornea, but these procedures are invasive and time-consuming and therefore used infrequently in busy clinics.

Optical coherence tomography angiography (OCTA) is an expedient functional extension of the OCT technique initially introduced in 1991. OCT is based on the interference principle to non-invasively acquire high-resolution images of biological tissues with near infrared light and an axial resolution of 1–2 μm^12^. OCTA reconstructs microvasculature in vivo down to the capillary level through the detection of moving particles (i.e., red blood cells) in tissue by assessing phase or amplitude differences from repeated OCT scans acquired at the same location. OCTA has many potential advantages over currently used anterior segment imaging techniques: First, it is preferred over FA and ICGA due to its non-invasive nature and dye-free application. Secondly, it is a patient-friendlier, and time- and cost-effective method without requiring certified technicians to perform the examination. However, current knowledge is limited and previous applications for the anterior segment, especially the cornea, few.

The purpose of this pilot study was to investigate the use of non-invasive anterior-segment optical coherence tomography angiography (AS-OCTA) for identifying and objectively quantifying corneal vascularization in high-risk eyes scheduled for keratoplasty and show its superiority to slit-lamp photography.

## Methods

This retrospective pilot study was conducted at the Department of Ophthalmology and Optometry at the Medical University of Vienna. All study investigations complied with the tenets of the Declaration of Helsinki. The study was approved by the Ethics Committee of the Medical University of Vienna (EK1295/2022) and informed consent was obtained from all subjects before inclusion in this study.

We included adult patients who presented to the tertiary clinic for corneal diseases between December 2020 and March 2022 and were scheduled for PK following a diagnosis of significant vision loss due to stromal scarring and vascularization in at least one corneal quadrant. These eyes were considered as “high-risk” eyes^5^ with regards to the planned PK. Defined exclusion criteria were missing records of OCTA images, or OCTA imaging results of insufficient quality as defined below, and the presence of only superficial corneal vascularization due to limbal stem cell deficiency.

At presentation, all study patients received a comprehensive anterior segment slit-lamp examination, their non-contact intraocular pressure (iCare IC100, Oy, Vantaa, Finland) was measured, and a biomicroscopical examination of the posterior segment in mydriasis (Mydriaticum 0.5%, Agepha Pharma, Senec, Slovakia) undertaken or, otherwise, an ophthalmic ultrasonography to grade vitreous or posterior segment pathology if media opacification obscured biomicroscopic fundus visualization. Evaluation of the anterior segment included identification of corneal opacification, scars and vascularization (number of affected corneal clock hours) as well as the presence of epithelial defects or ulcers/corneal melt.

The cornea of the study eye was imaged from limbus to limbus using slit-lamp color photography (SL 800, Carl Zeiss Meditec, Dublin, California, USA) focused on the corneal vascularization/scar/opacity (= region of interest, ROI). Ten-fold and 16-fold magnifications were used with diffuse illumination settings.

Color images were graded for quality from 0 to 3 as previously suggested: 0, no focus on ROI; 1, poor focus on ROI, no details of corneal scar/vessel; 2, acceptable focus on ROI, identifiable corneal scar/vessel; 3, very good or excellent focus on ROI, details of corneal scar and vessels evident^13^. FIJI (ImageJ 2.0.0, imagej.net) was used to delineate the corneoscleral junction (i.e. the limbus) in all images of sufficient quality (defined as ≥ grade 2), with image details beyond the limbal borders masked in black. Two widely used vessel segmentation methods, proven efficient for retinal vessels^14,15^, were used to segment CV in slit-lamp photographs. The transformations were executed in Python. The approach involved utilizing the green channel of the slit-lamp photograph for the examination and segmentation of vessel structures as CV exhibit greater contrast in the green channel compared to the red and blue channels, which tend to be noisy and display lower vessel contrast. As a first approach, we employed the morphological Hessian-Based method to segment and denoise CV using region-based Otsu Thresholding^15^. Secondly, CV in slit-lamp photographs were segmented using the established Gabor Filter with experimentally selected parameters that proved most effective for the segmentation of corneal vessels θ = from 0 to 170 degrees every 10 degrees then with the maximum, kernel size = (2,2), σ = 5, and λ = 0.5^14,16^. To enhance the result, histogram equalization techniques (i.e. the Contrast Limited Adaptive Histogram Equalization (CLAHE) operator) were employed to stretch the gray level values of low-contrast images.

Two independent investigators graded comparability between slit-lamp photographs and AS-OCTA images of the same eye as 1, comparable visualization of vascular details between imaging modalities, 2, superior visualization of vascular details in AS-OCTA images, 3, superior visualization of vascular details with slit-lamp photography.

### Anterior segment optical coherence tomography angiography (AS-OCTA):

Anterior segment OCTA was performed using a swept-source OCT system (Plex Elite 9000, Carl Zeiss Meditec, California, USA) equipped with a 10-diopter optical adaptor lens for anterior segment applications. The instrument achieves an optical axial resolution of approximately 6.3 μm in tissue by generating 100,000 A-scans per second. Non-invasive imaging was performed using 3 × 3-mm and 6 × 6-mm scan patterns given for retinal imaging that correspond to approximately 6 × 6-mm and 12 × 12-mm scan patterns in the cornea. Proof of quality of the images was obtained using the signal strength (SS) index given by the inbuilt software (version 2.0.1.47652) ranging from 0 to 10. We only included images with SS ≥ 7, without significant motion artifacts or blur for the further analyses in this study.

The segmentation algorithm of the inbuilt software was designed for the posterior segment of the eye; therefore, the inbuilt automated segmentation was prone to fail and layer segmentation had to be manually corrected for each corneal AS-OCTA volume stack. Slabs were rearranged for all of the 300 B-scans to delineate the epithelial and endothelial surface, and, thereby, the corneal anterior and posterior limits. The inbuilt software was further used to measure the maximum CV depth (posterior border of the deepest flow signal from epithelial surface) and the CV depth (= ratio between CV thickness and total corneal thickness, %).

### Optical coherence tomography angiography (AS-OCTA) image analysis

FIJI was used for pre-analysis of the en-face AS-OCTA images. In a first step, the corneoscleral junction was delineated and defined as the limbus in 6 × 6-mm AS-OCTA scans. In 3 × 3-mm AS-OCTA scans, the scan was focused on the circumscribed CV only, so this step was negligible. Afterwards, the scleral complementary flow signal and corneal background flow information surrounding the CV lesion were masked in black to avoid background noise influencing subsequent vessel measurements.

We further used Angiotool (0.6a, National Cancer Institute, USA), which is an open-source, validated and reproducible software for quantifying vascular networks^17–19^.

Semi-automated determination of CV-related parameters was achieved with the same customized settings for each patient regarding threshold, vessel thickness and removal of small particles in the outcome images. Angiotool applies a multi-scale Hessian analysis approach with smoothing using a recursive Gaussian filter to identify and segment vessels, followed by skeletonizing and skeleton analysis of the “explant area (EA)”, which is the total area of the vascular complex (CV lesion size, mm^2^). The software then computes several morphologic and spatial measures of a vessel including “total vessel length (TVL)”, which is the sum of all vessels defined as the distance between two junctions or endpoints (mm), “total number of junctions (TNJ)”, which is the total number of vessel junctions inside the explant area, “junction density (JD)”, which is the number of junctions per unit area (n/mm^2^), “total number of endpoints (TNEP)”, which is the number of open-ended vessel segments, “lacunarity, (L)”, which is the index for vascular structural non-uniformity, where higher values reflect more heterogenous vasculature with lower values more homogeneous vasculatures, “vessel area (VA)”, corresponding to the area of the AS-OCTA flow information inside the explant area (mm^2^), and “vessel density (VD)”, which is the percentage of segmented vessel area inside the explant area (%).

Automated vessel detection and vessel parameter quantification was not attempted in slit-lamp photographs as Angiotool software is not applicable and available for this modality.

### (Lymph-)angioregressive treatment prior to keratoplasty

At the discretion of the ophthalmologist charged with the patient’s care, high-risk eyes with CV scheduled for PK received (lymph-)angioregressive treatments prior to keratoplasty. These included peripheral crosslinking^11^ and fine needle-diathermy combined with bevacizumab^9^.

### Statistical analysis

For quantitative variables, we report the mean ± standard deviation (SD). For qualitative variables, we report the absolute and relative frequencies.

## Results

We included a total of twenty-six patients (mean age ± SD: 51 ± 16 years, 8 female) in this pilot study. Twenty-nine high-risk eyes due to significant CV (right eye = 12, left eye = 11, both eyes = 3) of these 26 patients were scheduled for PK. All of them showed sufficient image quality in AS-OCTA imaging (SS ≥ 7 as pre-defined) and were therefore included for further analyses.

The (presumed) diagnosis and cause of CV in the eyes of this cohort was trauma (n = 3), combustion (n = 1), chemical burn (n = 4), neurotrophic keratopathy (n = 3), infectious keratitis (acanthamoeba, n = 3; herpes simplex virus, n = 7; pseudomonas aeruginosa, n = 1; fungi, n = 1), limbal dermoid (n = 1), keratoconus (n = 2), congenital keratopathy (n = 1), and infantile cystinosis (n = 2). Fourteen eyes had undergone prior keratoplasty.

**Figure 1:**
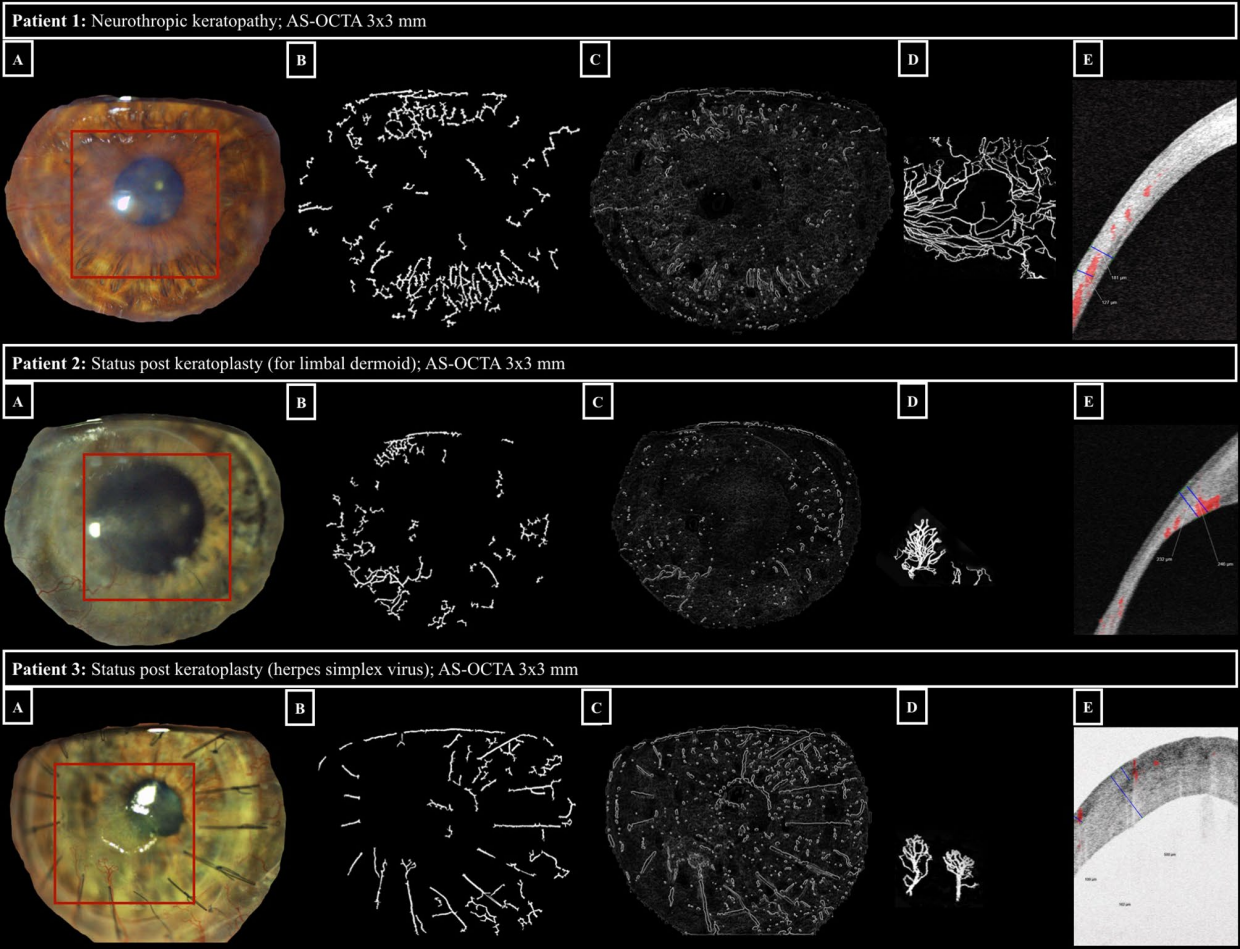
Comparison of slit-lamp photography and AS-OCTA imaging for corneal vascularization. Examples of eyes (respective image rows) with corneal vascularization imaged with slit-lamp photography (panels A: visualized corneal area delineated with Fiji, see Methods, region of interest imaged by AS-OCTA highlighted with a red rectangle); panels B: segmentation of corneal vascularization using the morphological hessian-based method; panels C: segmentation of corneal vascularization using Gabor filtering), and 3 × 3-mm AS-OCTA (panels D: AS-OCTA en-face scan after post-processing in Fiji: capillary flow area delineated in white; panels E: AS-OCTA cross-sectional scan showing a vessel flow signal in red with its marked (blue) deepest extension in relation to total corneal thickness).

Of the 29 eyes included based on image quality criteria in AS-OCTA, only 20 (70%) showed sufficient image quality (defined as ≥ grade 2) on slit-lamp photographs. Both graders scored comparable visualization of vascular details between imaging modalities (slit-lamp photography and AS-OCTA) in only one of the study eyes, whereas the graders agreed that AS-OCTA images of visualization of vascular morphology was superior to slit-lamp photographs in 28 of 29 eyes (97%). Figure 1 shows three representative comparisons of CV visualization on slit-lamp photographs versus AS-OCTA images (en-face and cross-sectional 3 × 3-mm AS-OCTA scans, respectively). It highlights that the use of colour thresholding in slit-lamp photographs, irrespective of the vessel segmentation method used, did not enhance vessel detection when compared to the conventional subjective evaluation of slit-lamp images.

**Figure 2:**
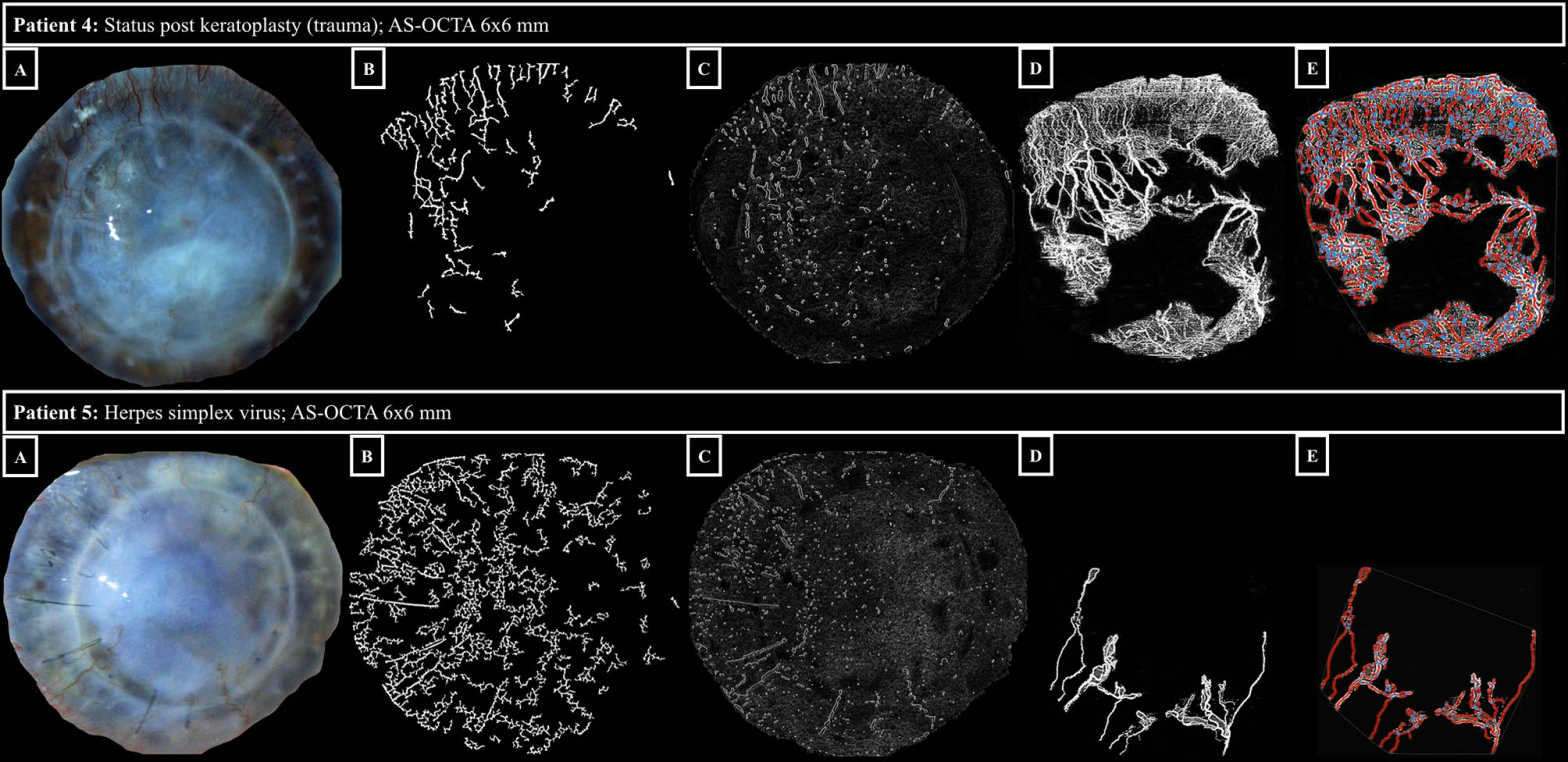
Vessel segmentation using Angiotool software. The rows represent the respective patient cases. Panels A show slit-lamp photographs, panels B show segmentation of corneal vascularization (morphological hessian-based method on slit-lamp photographs), panels C show segmentation of corneal vascularization (Gabor filtering on slit-lamp photographs), panels D native OCTA 6 × 6-mm en-face scans (effectively covering 12 × 12 mm on the corneal surface, see Methods) with the capillary flow area in white, and panels E OCTA 6 × 6-mm en-face scans after Angiotool software segmentation with delineated vessels (red), and vessel branching/end points (blue). See the attached Table 1 below for corresponding vessel parameters of patient 4 and 5 (ID 4 and 5, respectively). In patient 5, the vessels in the upper sectors of the cornea are not captured by the OCTA algorithm, which may be due to slow erythrocyte flow.

The mean signal strength of the AS-OCTA volumes included was 8 ± 1. After manual segmentation correction in all of the 300 B-scans of each AS-OCTA volume stack and background flow blackening in Fiji, Angiotool software was able to detect the capillary details and successfully measure the predefined vascular parameters in all study eyes. Figure 2 illustrates examples of native AS-OCTA images after post-processing and the corresponding AS-OCTA images after Angiotool software skeletonization and segmentation with the associated vascular parameter outcomes (6 × 6-mm AS-OCTA scans).

**Table 1:** Angiotool-derived vessel parameters of patient 4 and 5.

ID	Explant area (mm^2^)	Vessel area (mm^2^)	Vessel density (%)	Total number of junctions	Junction density (%)	Total vessel length (mm)	Total number of end points	Mean lacunarity
4	86.5	30.4	35.2	583	6.7	269.3	300	0.3
5	86.2	11.3	13.2	70	0.8	75	32	1.3

**Figure 3:**
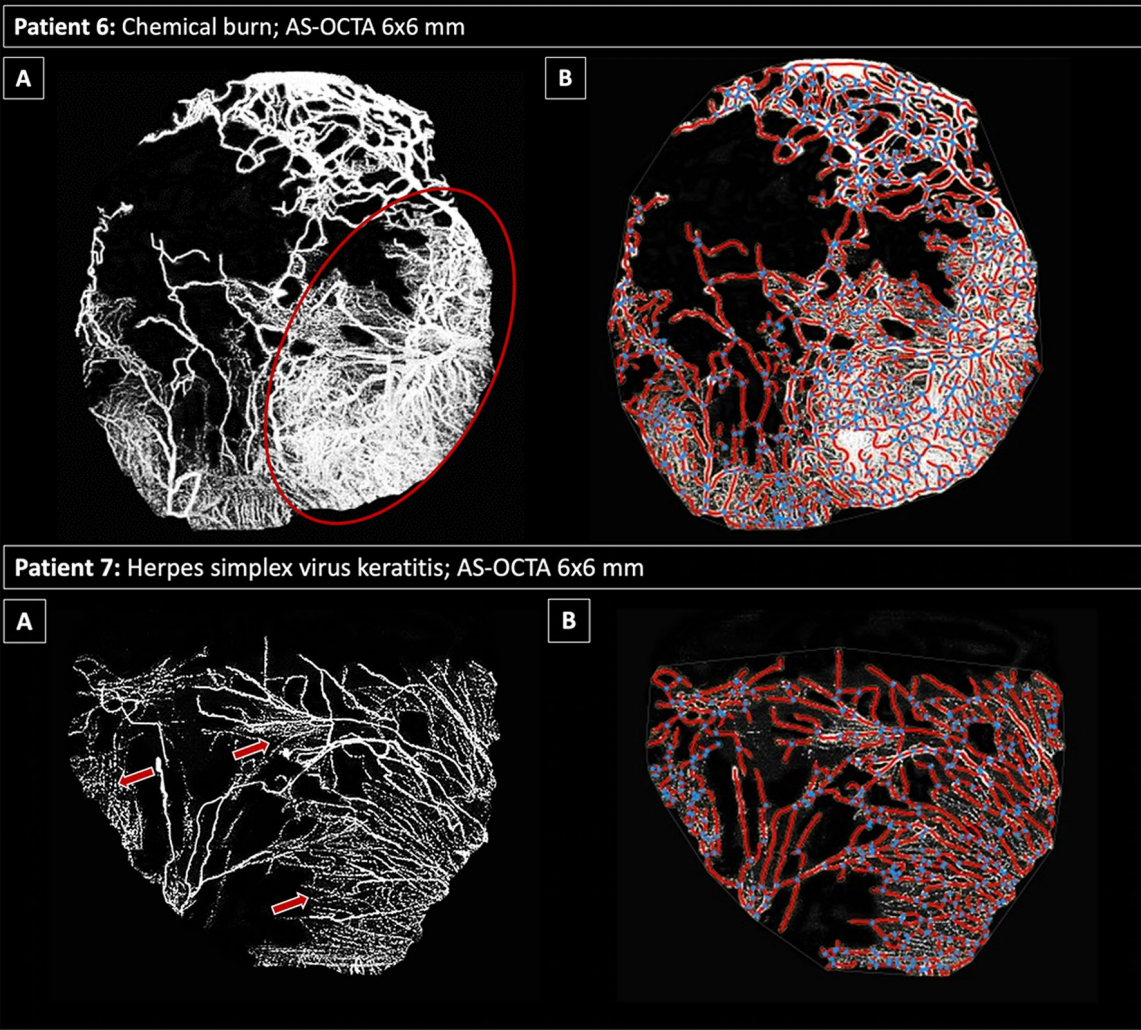
Limitations in vessel segmentation using Angiotool software. The rows represent the respective patient cases. Top row: Panel A shows the native AS-OCTA 6 × 6-mm en-face scan (effectively covering 12 × 12 mm on the corneal surface, see Methods) with the capillary flow area in white with a dense vascularized area (red ellipse); Panel B illustrates the corresponding AS-OCTA scan after Angiotool segmentation with vessels in red and branching/end points in blue. In the densely vascularized area corresponding to the red ellipse (top row, panel **A**), the software fails to discriminate between individual capillary channels. Bottom row: Panel A shows the native AS-OCTA 6 × 6-mm en-face scan with fine capillaries marked by red arrows; these capillary details are not recognized by the Angiotool software algorithm (Panel **B**). See the attached Table 2 below for corresponding vessel parameters of patient 6 and 7 (ID 6 and 7, respectively).

The limitations of the Angiotool software measurements become evident in the presence of dense capillary invasion with or without concomitant scarring, where the algorithm fails to precisely delineate the individual fine vessel segments and/or branching patterns (Fig. 3).

**Table 2:** Angiotool-derived vessel parameters of patient 6 and 7.

ID	Explant area (mm^2^)	Vessel area (mm^2^)	Vessel density (%)	Total number of junctions	Junction density (%)	Total vessel length (mm)	Total number of end points	Mean lacunarity
6	127.9	50.9	39.8	697	5.5	388.3	268	0.2
7	77.6	27.0	34.8	318	4.1	221.7	208	0.3

**Figure 4:**
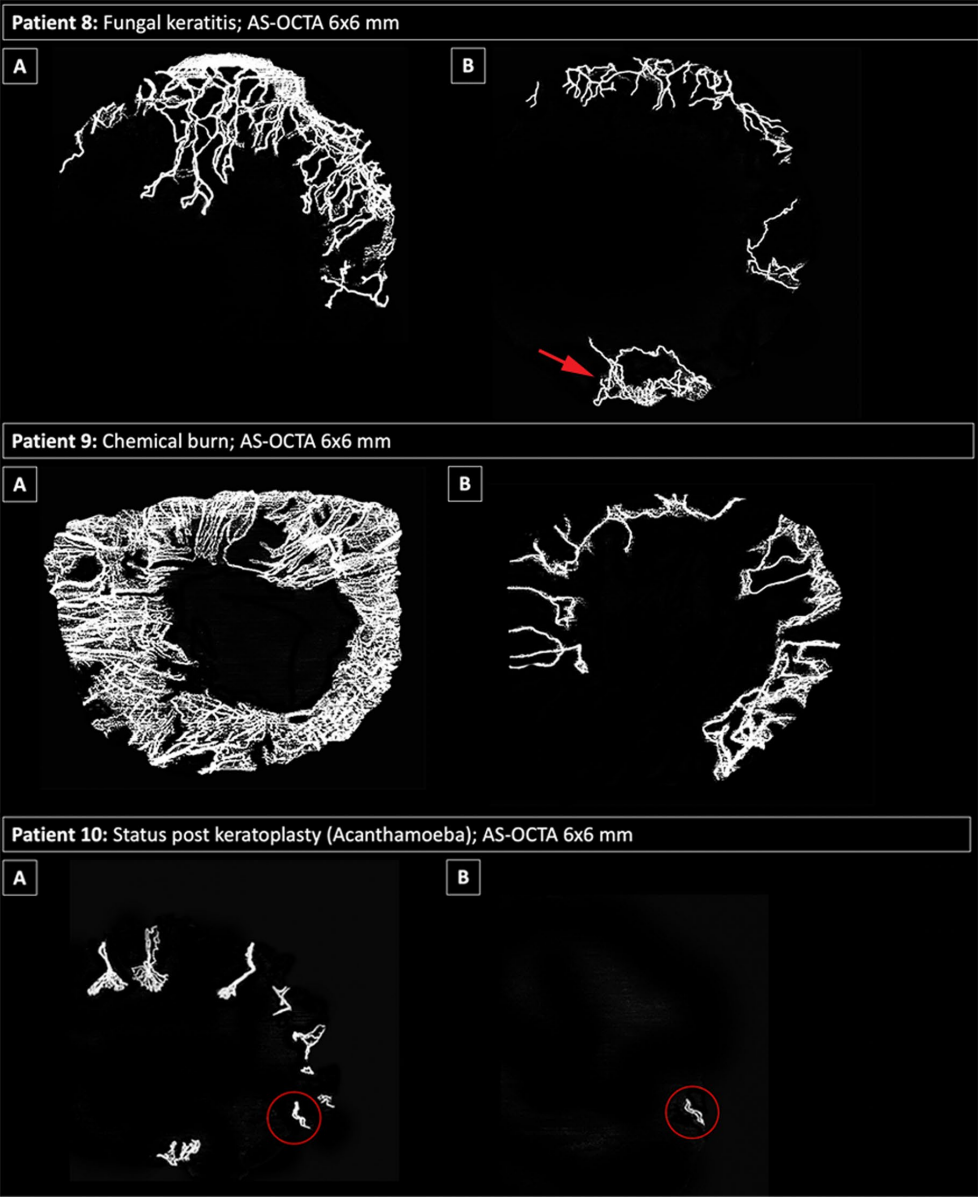
AS-OCTA imaging for monitoring of treatment effects. Examples of eyes (respective image rows) treated for corneal vascularization. Top row: Patient 8 before treatment (panel **A**) and 1 month after angioregressive treatment with selective superior peripheral crosslinking (panel **B**). Note that the inferior peripheral cornea was not included in the treatment zone, where new vessels had grown at follow-up (red arrow). Middle row: Patient 9 before treatment (panel A) and 1 month after subconjunctival injection with bevacizumab (panel **B**). Bottom row: Patient 10 before treatment (panel **A**) and 1 month after circular peripheral crosslinking combined with a 3-month regimen of topical bevacizumab (panel B), with almost total regression of vessels visualized in AS-OCTA—a small vessel loop remains inferiorly (red circle).

Sixteen of the 29 eyes received (lymph-)angioregressive treatment during the study inclusion time span. Figure 4 illustrates the response to (lymph-)angioregressive therapy in three of these eyes as monitored with AS-OCTA.

## Discussion

This pilot study explored the use of anterior-segment optical coherence tomography angiography imaging for the quantitative visualization of corneal vascularization.

The major findings were that AS-OCTA imaging 1. allows a detailed and more expedient visualization of CV than slit-lamp photography, 2. may be applied in combination with a readily available segmentation software for objective and quantitative vascular profiling, and 3. can be successfully used as a monitoring tool during angioregressive treatment for CV.

A comprehensive meta-analysis by Bachmann et al. on a total of 24,944 grafts undergoing keratoplasty showed that the presence of pathologic CV increases the risk of graft failure and rejection with a pooled risk ratio of 1.32 (95% confidence interval (CI) 1.15–1.49) for graft failure and 2.07 (95% CI 0.98–3.15) for graft rejection^5^. The results of their study further support those of the largest graft registry to-date, which showed that both graft failure and rejection risk increase when a higher number of corneal quadrants are affected by CV prior to keratoplasty^20^.

Cornea specialists around the world therefore agree on the urgent need for effective treatments to precondition high-risk eyes in order to achieve regression of lymphatic and blood vessels prior to PK, and prevent re-angiogenesis in the post-operative period. In addition, we here propose that these therapeutic options will only be valuable if monitoring of CV progression with rapid, repeatable and objective imaging techniques is assured.

Slit-lamp photography uses a white light source with the option of different additional filters and illumination patterns that is easily accessible and yet has limited reproducibility and image quality as an effect of lighting, and device- or patient-dependent factors. Figures 1 and 2 show that in the presence of corneal haze (Fig. 1, patient 1 and 2) or scarring/opacification (Fig. 2, patient 4 and 5), fine capillary details are obscured, even in high-quality slit-lamp photographs. Moreover, these photographs are known to favor venous vessels, being more numerous and of larger diameter, over the smaller, usually more deeply located, arteries^13^. OCT signals, on the other hand, easily penetrate even opaque corneal tissue. As a result, en-face AS-OCTA images can reveal microvascular detail to a much greater extent than slit-lamp photographs (Figs. 1 and 2). Even more importantly, AS-OCTA is a three-dimensional imaging technique, offering the opportunity to assess the precise depth of erythrocyte flow signals alongside the total vascular tree (Fig. 1, panels C). This major advantage of AS-OCTA imaging is invaluable when planning (lymph-)angioregressive treatment regimens for forecasting potential endothelial damage prior to peripheral crosslinking or estimating the ideal treatment depth when using fine-needle diathermy for occlusion of CV.

Even if traditional angiographic techniques including FA and ICGA reach up to three- to fourfold more visibility of corneal blood vessels than slit-lamp photography, and ICGA additionally achieves excellent vessel delineation in the presence of stromal scarring^13,21^, these techniques are time consuming, invasive and potentially associated with systemic adverse events. They are therefore infrequently performed for the anterior segment compared with the posterior segment in clinical practice. AS-OCTA on the other hand, can serve as a feasible, non-invasive and repeatable alternative to invasive dye-based angiography with CV area measurements comparable to ICGA results, as has been shown in animal models^22,23^ and small clinical pilot studies^24,25^. However, a standardized quantitative evaluation of CV with AS-OCTA has not yet been established, and previous series have all relied on customized vascular metrics and CV grading scores.

For the purpose of this study, we hypothesized that the Angiotool software, already successfully applied for choroidal neovascularization quantification^19,26^, can provide a solid segmentation tool for a reliable, semi-automated vascular quantification with diverse metrics for corneal vascularization too. After quality control, manual segmentation correction, and binarization of AS-OCTA images, the Angiotool software algorithm recognized blood vessel channels and branching/end points in all of the eyes included in this study (see Fig. 2), but certain limitations of the algorithm must be considered: Fig. 3 demonstrates that the algorithm fails to discriminate individual capillaries of small diameter or if localized within dense vascular networks. Furthermore, vessels with slow erythrocyte flow, below the slowest detectable flow threshold of the OCTA device, may not be detected by the algorithm (see Fig. 2 patient 5).

Moreover, we applied AS-OCTA imaging to monitor three eyes during angioregressive treatment protocols in this study, and therefore provide the first evidence for its major advantage as a monitoring tool. We found it is not only suitable for study purposes but also for clinicians caring for patients in busy clinics to precisely define the true extent of corneal vascular invasion prior, during and after treatment (see Fig. 4). Today, there is a wide range of AS-OCTA systems available on the market, and inter-device differences due to diverse software algorithms cannot be ruled out. It is therefore important to consider that these devices should not be used interchangeably.

This study is limited by its small sample size due to the nature of a pilot study. Patients did not undergo additional invasive angiography, as this study was not designed to investigate comparability between AS-OCTA and invasive angiography techniques, which has already been a focus of previous studies^27^.

In conclusion, we present the results of a pilot study that promote AS-OCTA as a precise, non-invasive diagnostic tool for high-resolution quantification of corneal vascularization not only superior to slit-lamp photography but also capable of assessing novel vascular metrics. AS-OCTA imaging may serve as an easily accessible, cost-effective technique for monitoring angioregressive preconditioning therapies in high-eyes before keratoplasty, which requires further investigation in future longitudinal clinical trials.

## Data Availability

All data is available at the Department of Ophthalmology at the Medical University of Vienna. The datasets used and/or analyzed during the current study available from the corresponding author on reasonable request.
